# Detection of early renal perfusion changes by contrast‐enhanced ultrasonography in dogs with preclinical myxomatous mitral valve disease

**DOI:** 10.1111/vru.13459

**Published:** 2024-11-12

**Authors:** Saho Kamata, Tomoya Morita, Masahiro Yamasaki

**Affiliations:** ^1^ Laboratory of Veterinary Small Animal Internal Medicine Cooperative Department of Veterinary Medicine Faculty of Agriculture, Iwate University Morioka Japan

**Keywords:** canine, intrarenal Doppler ultrasonography, kidney, mitral regurgitation, right ventricular dysfunction

## Abstract

Since the prognosis of myxomatous mitral valve disease (MMVD) varies, its characterization is clinically relevant, and renal impairment has been identified as one of its associated factors. Intrarenal Doppler ultrasonography (IRD), an intrarenal hemodynamic assessment method, is useful for predicting cardiac‐ and renal‐related death but cannot detect early changes in dogs with preclinical MMVD. Contrast‐enhanced ultrasonography (CEUS), another intrarenal hemodynamic assessment method, may identify earlier changes; however, renal perfusion evaluations using CEUS have not yet been performed on dogs with MMVD. We hypothesized that CEUS detects changes earlier than IRD in dogs with preclinical MMVD. This prospective, cross‐sectional study examined renal perfusion in dogs without cardiac disease and preclinical MMVD dogs of different American College of Veterinary Internal Medicine stages using CEUS and compared it with IRD indices. Twenty‐three dogs with MMVD (ten stage B1 and thirteen stage B2) and 12 control dogs without cardiac disease were included. The rise times of the renal cortex and medulla were measured from a time‐intensity curve. The rise time of the cortex was longer in dogs with stage B2 MMVD than in control dogs, while that of the medulla was shortened in the right ventricular dysfunction group in stage B2. No changes were observed in IRD indices (the resistance index and venous impedance index). In conclusion, CEUS detected changes in renal perfusion in dogs with preclinical MMVD even when IRD indices remained unchanged, suggesting the utility of CEUS in evaluations of renal perfusion in MMVD dogs.

## 1 INTRODUCTION

Myxomatous mitral valve disease (MMVD) is the most common heart disease in dogs. It is characterized by a long preclinical period, and its prognosis varies.^1^ Therefore, the prognostic characterization of MMVD is a subject of considerable clinical interest.^1^, ^2^ Renal function is one of the important prognostic factors in dogs with MMVD, and survival times were found to be shorter in dogs with both MMVD and chronic kidney disease (CKD) than in those with MMVD alone.^3^ In heart failure human patients, a low stroke volume decreased renal perfusion, while right ventricular (RV) dysfunction caused renal congestion accompanied by an increased renal interstitial pressure, leading to renal dysfunction.^4^, ^5^, ^6^, ^7^ These mechanisms change intrarenal blood flow. Therefore, the evaluation of intrarenal hemodynamics has attracted attention.

Intrarenal Doppler ultrasonography (IRD), which is evaluated in interlobar vessels, is one of the methods used to assess intrarenal hemodynamics. Intrarenal venous flow may be affected by both central venous hemodynamics and reduced renal parenchymal compliance related to renal congestion; therefore, it may be useful for assessing renal congestion and predicting poor outcomes in human patients with heart failure.^8^, ^9^, ^10^, ^11^ We previously reported that an increase in the venous impedance index (VII), which is obtained from intrarenal venous flow, was associated with right atrial dilation and RV dysfunction assessed by echocardiography.^12^ Furthermore, increased VII was related to a poor prognosis in dogs with MMVD.^13^ Since the median survival of dogs with increased VII was very short (190 days; 95% confidence interval, 7–292 days), the early detection of intrarenal hemodynamic abnormalities using IRD was not possible.^13^

Contrast‐enhanced ultrasonography (CEUS) is another method for evaluating intrarenal hemodynamics. It is an imaging technique that uses microbubble‐based contrast agents to visualize the perfusion of microvascular beds. The assessment of intrarenal hemodynamics by CEUS has two possible advantages: (1) the assessment of peripheral vessels blood flow rather than the interlobar vessels by IRD, resulting in the early detection of abnormalities in intrarenal hemodynamics, and (2) the separate evaluation of cortical and medullary blood flow. In a rat model of hypertensive heart failure, Chiba et al.^11^ reported a relationship between increases in renal interstitial pressure and decreases in medullary perfusion evaluated by CEUS. Furthermore, Komuro et al.^14^ demonstrated that renal perfusion was reduced in both the cortex and medulla in heart failure human patients and that only cortical perfusion improved after treatment. These findings suggest that the behaviors of cortical and medullary perfusion differ during heart disease, and thus, an evaluation of medullary perfusion is important. Since CEUS has the capacity to assess the cortex and medulla separately, it may detect differences in cortical and medullary perfusion as MMVD progresses.

Contrast‐enhanced ultrasonography identified changes in renal perfusion in renal disease and ischemic renal disease models in dogs^15^, ^16^, ^17^; however, it has not yet been conducted on dogs with MMVD. We hypothesized that CEUS detects changes in renal perfusion earlier than IRD, even in preclinical MMVD. Therefore, the present study investigated renal perfusion in dogs without cardiac disease and preclinical MMVD dogs of different American College of Veterinary Internal Medicine (ACVIM) stages using CEUS, and the study examined the impact of RV function contributing to renal congestion on perfusion indices and elucidate the relationships between perfusion indices and factors affecting intrarenal hemodynamics.

## 2 MATERIALS AND METHODS

### 2.1 Dogs and study design

This was a prospective cross‐sectional study. All MMVD and control dogs recruited in the present study were referred to Iwate University Veterinary Teaching Hospital between May 2022 and September 2023. Informed consent was obtained from all owners. This study was approved by the Animal Research Committee of Iwate University (no. A202235).

Inclusion criteria for preclinical MMVD dogs were the presence of a left apical systolic murmur, the presence of mitral regurgitation on Doppler echocardiography in conjunction with mitral valvular lesions (i.e., thickening or prolapse of the mitral valve leaflets). Dogs underwent thoracic radiography and echocardiography and were classified as stages B1 and B2 based on the ACVIM consensus statement guidelines.^18^ Dogs with current or previous evidence of cardiogenic pulmonary edema and/or pulmonary venous congestion were excluded. Dogs with known clinically relevant systemic or organ‐related diseases, such as any neoplasia, adrenal dysfunctions, thyroid dysfunctions, and stage 4 CKD confirmed by physical examination, blood tests, radiography, or ultrasound, were also excluded. Chronic kidney disease was diagnosed according to the classification of the International Renal Interest Society, and dogs with a serum creatinine concentration >5.0 mg/dL were diagnosed with stage 4 CKD.^19^ The probability of pulmonary hypertension (PH) was examined using echocardiography according to the ACVIM consensus statement guidelines.^20^

Control group inclusion criteria were client‐owned dogs and laboratory Beagles older than 7 years, the absence of a heart murmur, and no evidence of heart disease on echocardiography. Dogs with clinically relevant systemic or organ‐related diseases, including any neoplasia and adrenal and thyroid dysfunctions, and receiving medication that affects the cardiovascular system, such as steroids, were excluded. Dogs with stage 2 CKD diagnosed with a serum creatinine concentration >1.4 mg/dL were also excluded.^19^

### 2.2 Echocardiographic measurements

The echocardiographic examination was performed by one echocardiographer (TM) with 13 years of experience in echocardiography using an ultrasound unit (Aplio a Verifia, Canon Medical Systems Corporation) with a sector transducer frequency ranging between 2.8 and 6.2 MHz (PST‐50BT, Canon Medical Systems Corporation). A stand‐off pad was not used. Acoustic gel was applied to the skin. Dogs were positioned in right and left lateral recumbency. An electrocardiogram trace (lead II) was recorded simultaneously with echocardiographic imaging.

The left ventricular end‐diastolic diameter was measured from M‐mode echocardiography with a right parasternal short‐axis view. The normalized left ventricular end‐diastolic diameter was calculated using the following equation^21^:

$$\begin{array}{ccc} & & \mathrm{Normalized}\;\mathrm{left}\;\mathrm{ventricular}\;\mathrm{enddiastolic}\;\mathrm{diameter}=\mathrm{Left}\;\mathrm{ventricular}\\ & & \;\mathrm{enddiastolic}\;\mathrm{diameter}/{\left(\mathrm{Body}\;\mathrm{weight}\right)}^{0.294}. \end{array}$$

Left atrium and aorta root diameters were obtained with a right parasternal short‐axis view, and the left atrium to aorta root diameter ratio was calculated. Measurements of transmitral early diastolic flow velocity were performed with pulsed‐wave Doppler with a left apical four‐chamber view. The peak tricuspid regurgitation velocity was measured from the echocardiographic view, which provided the highest velocity. To assess the RV function, the RV Tei index was calculated using tissue Doppler as the sum of the isovolumic time derived by subtracting the peak systolic tricuspid annular velocity duration from the time from the end of the late diastolic tricuspid annulus velocity to the onset of the early diastolic tricuspid annulus velocity.^22^ The stroke volume was calculated by multiplying the time velocity integral of pulmonary Doppler flow by the pulmonary artery cross‐sectional area.^23^ In the left parasternal short‐axis view, pulmonary artery Doppler flow was recorded and manually traced to obtain the time velocity integral, and the pulmonary artery diameter was measured by tracing the pulmonary artery luminal dimension. The pulmonary artery cross‐sectional area was calculated using the following formula:

$$\mathrm{Cross}\;\mathrm{sectional}\;\mathrm{area}=\pi \times {\left(\mathrm{Pulmonary}\;\mathrm{artery}\;\mathrm{diameter}/2\right)}^{2}.$$

The stroke volume index was obtained by dividing stroke volume by body surface area.^23^ Body surface area was calculated as follows:

$$\mathrm{Body}\;\mathrm{surface}\;\mathrm{area}\;=10.1\times \frac{{\left(\mathrm{Body}\;\mathrm{weight}\;\right)}^{\frac{2}{3}}}{10^{4}}.$$

### 2.3 Laboratory data

Blood samples were collected by venipuncture into serum tubes. The serum was separated by centrifugation at 3000 rpm at room temperature for 15 min and used to measure blood urea nitrogen and creatinine levels.

The glomerular filtration rate was measured by inulin clearance. Following the intravenous administration of 100 mL/kg of inulin (Inulead, Fuji Yakuhin Co., Ltd.) through the cephalic vein, blood was collected after 2 and 3 h. The glomerular filtration rate was measured at a commercial laboratory (FUJIFIRM VET Systems). The normal was greater than >55.2 mL/min/m^2^.

### 2.4 Contrast‐enhanced ultrasonography

Contrast‐enhanced ultrasonography was performed by one observer (T.M.) with 13 years of experience in ultrasonography using the same ultrasound system as echocardiography with a 7.2–14.0 MHz linear probe (PLT‐1204BT, Canon Medical Systems Corporation). A stand‐off pad was not used. Acoustic gel was applied to the skin. Dogs were positioned in the right lateral recumbency, and a longitudinal view of the left kidney was imaged. The CEUS was performed only once on the left kidney in unsedated dogs. A contrast agent (Sonazoid, GE Healthcare) was administered as an intravenous bolus in a dosage of 0.01 mL/kg and flushed with 2 mL saline from an IV catheter placed in the cephalic vein. Image acquisition was initiated at the start of the contrast agent injection, and images were recorded for 30 s. The mechanical index was adjusted at 0.20, the gain was 80, the dynamic range was 60 dB, and the frame rate was 19 fps. The focal depth was placed in the middle of the left kidney.

### 2.5 Contrast‐enhanced ultrasonography analysis

**FIGURE 1:**
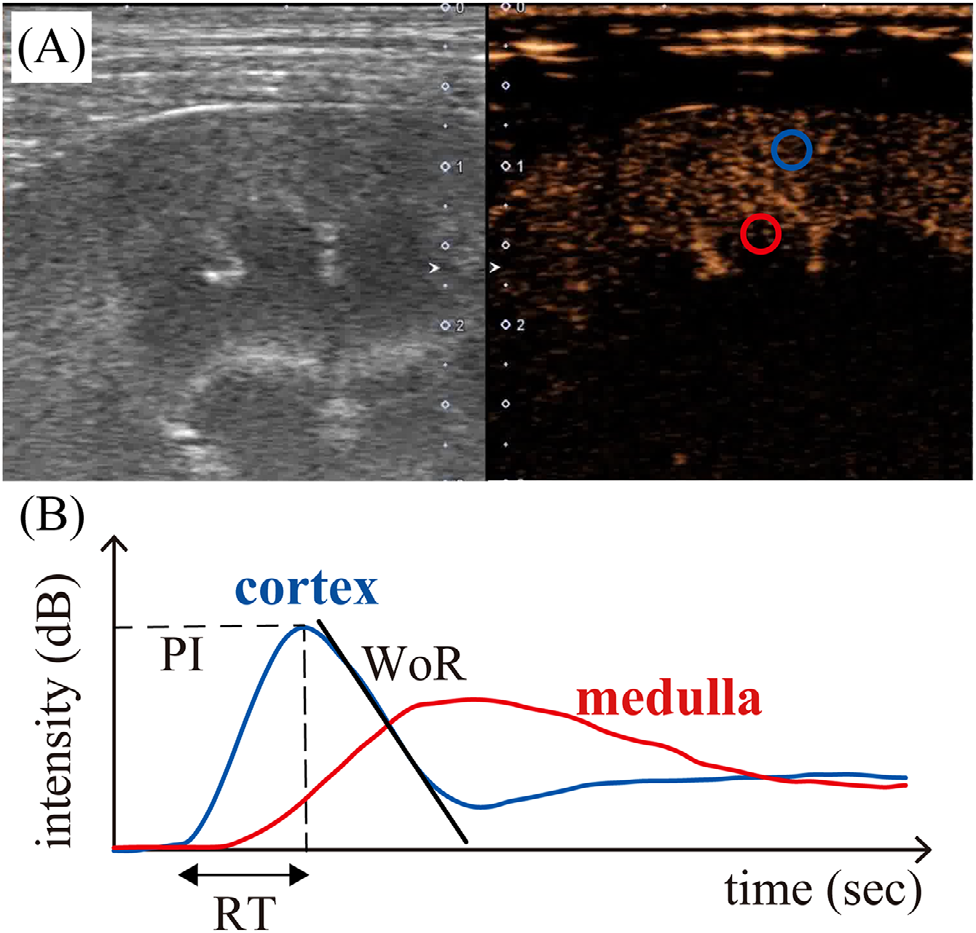
Contrast‐enhanced ultrasonography of a representative dog (4‐year‐old female beagle). A, The left panel shows the B‐mode image and the right panel shows the image seven seconds after the injection of sonazoid. Regions of interest were 5 × 5 mm in the renal cortex (blue circle) and as large as possible to fit within the renal medulla (red circle). B, Time‐intensity curve with smoothing of the cortex (blue curve) and medulla (red curve). The X‐axis represents the time in seconds (s), while the Y‐axis shows intensity (dB). The rise time was defined as the time from the initial rise to the peak. The wash‐out rate was defined as the downward slope as the enhancement decreases from 95% to 85%. Peak intensity was defined as the maximum enhancement. PI, peak intensity; RT, rise time; WoR, wash‐out rate.

A quantitative analysis of CEUS images was performed using analysis software (ImageJ, US National Institutes of Health). Three circle‐shaped regions of interest (ROI) of 5 × 5 mm in the renal cortex were drawn (Figure 1A). Three circle‐shaped ROIs as large as possible to fit within the renal medulla were drawn (Figure 1A). The position of the ROI was manually adjusted if the position of the kidney shifted due to breathing or if it overlapped with the interlobar artery or arcuate artery. The intensity of the three ROIs was measured at 0.5 s intervals. Time‐intensity curves of the cortex and medulla were generated from the average intensity obtained in the three ROIs, respectively. The time‐intensity curve was smoothed using the Savizky‐Golay method by numerical analysis software (MATLAB R2023a, Mathworks Inc.).^24^ The rise time (RT; s), which reflects perfusion velocity entering the kidney, the wash‐out rate (WoR), which reflects perfusion velocity exiting the kidney, and the peak intensity (PI; dB), which reflects perfusion volume were calculated (Figure 1B). Rise time was defined as the time from the initial rise to the peak; wash‐out rate was defined as the downward slope as the enhancement decreased from 95% to 85%, and peak intensity was defined as the maximum enhancement.

### 2.6 Intrarenal Doppler ultrasonography study

Intrarenal Doppler ultrasonography was performed by one observer (T.M.) with 13 years of experience in ultrasonography using the same ultrasound system and transducer as echocardiography. It was conducted with a simultaneous lead II electrocardiographic recording. A stand‐off pad was not used. Acoustic gel was applied to the skin. The IRD was performed in unsedated dogs. Dogs were positioned in the right lateral recumbency, and a longitudinal view of the left kidney was imaged. Interlobar arteries and veins were detected using color Doppler imaging (velocity range, 16.0–19.2 cm/s). The sample volume (2.0 mm) was set at the interlobar vessels. Pulsed‐wave Doppler waveforms of the interlobar arteries and veins were simultaneously recorded. The resistance index (RI) and VII were calculated as follows^25^:

$$\begin{array}{ccc} & & \mathrm{RI}=\left(\mathrm{Maximum}\;\mathrm{flow}\;\mathrm{velocity}-\mathrm{Minimun}\;\mathrm{flow}\;\mathrm{velocity}\right)/\\ & & \;\mathrm{Maximum}\;\mathrm{flow}\;\mathrm{velocity}\;\mathrm{from}\;\mathrm{interlobar}\;\mathrm{arteries}. \end{array}$$

$$\begin{array}{ccc} & & \mathrm{VII}=\left(\mathrm{Maximum}\;\mathrm{flow}\;\mathrm{velocity}-\mathrm{Minimum}\;\mathrm{flow}\;\mathrm{velocity}\right)/\\ & & \;\mathrm{Maximum}\;\mathrm{flow}\;\mathrm{velocity}\;\mathrm{from}\;\mathrm{interlobar}\;\mathrm{veins}. \end{array}$$

Measurements during the sinus rhythm were averaged over three cardiac cycles.

### 2.7 Statistical analysis

Statistical analyses were performed using a statistical analysis program (JMP version 14.0, SAS Institute Inc.). The normal distribution of data was confirmed by the Shapiro–Wilk test. Continuous data were presented as the mean ± SD for normally distributed data and as a median (interquartile range) for nonnormally distributed data. Two‐group comparisons for continuous variables were performed with the Student's *t*‐test and Wilcoxon test, and three‐group comparisons for continuous variables were conducted with Tukey's HSD test and Steel‐Dwass's test. Dogs with MMVD stage B2 were classified into a normal RV function group (RV Tei index < 0.62) and an RV dysfunction group (RV Tei ≥ 0.62) using the reference value of the RV Tei index.^22^

Repeatability and reproducibility evaluations were conducted using six laboratory Beagles. One dog was included in the control group, but the other five dogs were not included because they were younger than seven years. Intraday variability was assessed by performing CEUS three times a day with an interval of 3 h. Interobserver variability was evaluated by randomly extracting images recorded with intraday variability and performing the analysis independently with two analyses. Both analysts were blinded to each other's measurements. Coefficients of variation (CVs) were calculated by dividing each SD by the mean and were shown as a percentage. Based on previous studies on CEUS in humans and animals, CV < 25% was considered to be clinically acceptable.^26^

A *P*‐value < .05 was considered to be significant.

## 3 RESULTS

### 3.1 Study groups

Twenty‐three dogs with MMVD and 12 without cardiac disease (control group) were included. No dog was excluded due to no compliance. According to the ACVIM classification, ten dogs were classified as stage B1 and 13 as stage B2. The breed distribution consisted of Cavalier King Charles Spaniel (*n* = 4, 17.4%), Chihuahua, mixed breed, and Papillon (*n* = 3, 13.0% each), miniature schnauzer and toy poodle (*n* = 2, 8.7% each), and American cocker spaniel, beagle, chin, Pomeranian, Shi Tzu, and Shiba (*n* = 1, 4.3% each) for the MMVD group, and miniature dachshund (*n* = 6, 50%), mixed breed (*n* = 3, 25.0 %), toy poodle (n = 2, 16.7%), and beagle (*n* = 1, 8.3%) for the control group. Demographic data, laboratory data, and echocardiographic variables for the enrolled dogs are shown in Table 1. No significant differences were observed in age, body weight, heart rate, or systolic blood pressure between the control and MMVD groups. One dog in stage B1 and five dogs in stage B2 had a high probability of PH. At the time of enrollment, 15 dogs were receiving pimobendan, 6 an angiotensin‐converting enzyme inhibitor, and 4 furosemide.

**TABLE 1:** Comparison of clinical and echocardiographic variables in control, ACVIM stage B1, and ACVIM stage B2 groups.

	Control (*n* = 12)	Stage B1 (*n* = 10)	Stage B2 (*n* = 13)
Sex (male/female)	7/5	8/2	9/4
Age (year)	9 (8–13)	13 (10–13)	12 (11–13)
Body weight (kg)	6.0 (4.7–8.4)	6.8 (3.5–9.8)	5.2 (2.9–8.6)
Heart rate (bpm)	116 (96–162)	117 (99–172)	128 (100–170)
Systolic blood pressure (mmHg)	158 (151–169)	157 (145–167)	149 (142–156)
High probability of PH		1 (10%)	5 (38%)
Pimobendan		3 (30%)	12 (92%)
ACEI		1 (10%)	5 (38%)
Furosemide		1 (10%)	3 (23%)
BUN (mg/dL)	14.5 (11.1–22.1)	18.5 (12.8–21.0)	19.5 (10.3–34.8)
Creatinine (mg/dL)	0.81 (0.70–1.00)	0.70 (0.58–1.13)	0.80 (0.63–1.25)
GFR (mL/min/m^2^)^a^		45.1 (37.7–67.8)	40.1 (21.9–55.0)
LA/Ao	1.35 (1.24–1.43)	1.41 (1.38–1.58)	1.97 (1.76–2.11)^a^, ^b^
LVIDDN	1.33 (1.23–1.47)	1.47 (1.39–1.63)	1.83 (1.71–2.17)^a^, ^b^
Emax (m/s)	0.55 (0.43–0.63)	0.82 (0.66–0.93)	1.08 (0.84–1.21)^a^, ^b^
TRV (m/s)	2.74 (2.46–2.94)	2.49 (2.32‐2.72)	3.01 (2.64–3.27)
RV Tei index	0.55 (0.50–0.58)	0.47 (0.43–0.53)	0.64 (0.50–0.76)
SVI (L/min/m^2^)	24.4 (14.4–29.8)	24.2 (19.0–27.6)	25.1 (16.8–35.6)

*Note*: Data are expressed as medians (interquartile ranges).

Abbreviations: ACEI, angiotensin‐converting inhibitor; BUN, blood urea nitrogen; Emax, transmitral early diastolic flow velocity; GFR, glomerular filtration rate, LA/Ao, left atrium to aorta root ratio; LVIDDN, left ventricular internal dimension in diastole normalized for body weight; PH, pulmonary hypertension; RV, right ventricular; SVI, stroke vol

ume index; TRV, tricuspid regurgitation velocity.

^a^ The glomerular filtration rate was measured in fourteen dogs.

^b^ Differs significantly from the control (*P *< .05).

^c^ Differs significantly from ACVIM stage B1 (*P *< .05).

Blood urea nitrogen and creatinine levels did not significantly differ among the groups. Three dogs were classified as stage 2 CKD and none as stage 3. The glomerular filtration rate was measured in two in the control group (16.7%), six in the stage B1 group (60%), and six in the stage B2 group (46.2%). The glomerular filtration rate was decreased in four in stage B1 (66.7%) and five in stage B2 (83.3%). The stroke volume index did not significantly differ among the groups. In stage B2 dogs, seven (53.8%) were classified into the RV dysfunction group.

### 3.2 Repeatability and reproducibility evaluation

Intraday CVs for RT, WoR, and PI of the cortex were 11.1%, 15.9%, and 14.5%, and medulla were 20.0%, 21.8%, and 25.7%. Interobserver CVs for RT, WoR, and PI of the cortex were 11.4%, 27.6%, 16.8%, and medulla were 19.2%, 50.1%, and 39.4%. Only RT was used in this study because of the intraday and interobserver CV < 25%.

### 3.3 Intrarenal hemodynamics

**FIGURE 2:**
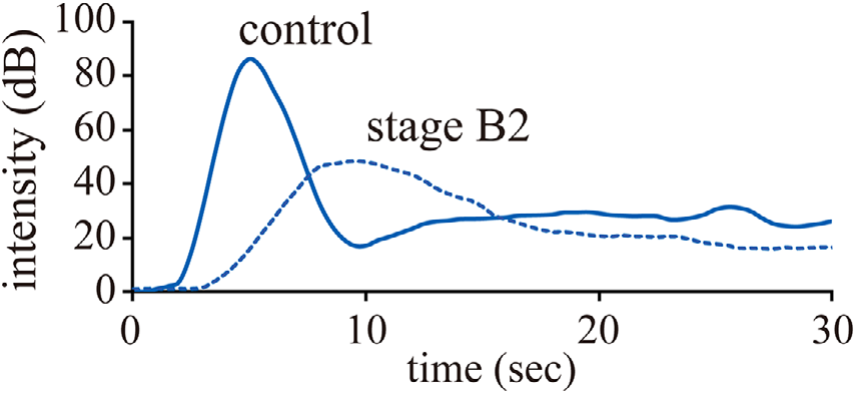
Time–intensity curve of the renal cortex obtained from a representative dog in a 9‐year‐old male miniature dachshund in the control group (blue solid line) and 10‐year‐old male Cavalier King Charles Spaniel in stage B2 (blue dashed line) groups. The rise time was slower in a dog with stage B2 than in a control dog. The X‐axis represents the time in seconds (s), while the Y‐axis shows intensity (dB).

The rise time of the cortex was longer in the ACVIM stage B2 group than in the control group, whereas that of the medulla did not significantly differ among the groups (Figure 2, Table 2).

**TABLE 2:** Mean ± SD of contrast‐enhanced ultrasonography and intrarenal Doppler ultrasonography indices in different groups.

	Control (*n* = 12)	Stage B1 (*n* = 10)	Stage B2 (*n* = 13)
CEUS			
Cortex RT (s)	3.9 ± 0.8	4.6 ± 0.9	5.0 ± 0.9^a^
Medulla RT (s)	8.1 ± 3.0	7.4 ± 2.1	7.0 ± 1.5
IRD^b^			
RI	0.64 ± 0.05	0.66 ± 0.09	0.71 ± 0.08
VII	0.24 ± 0.06	0.24 ± 0.05	0.24 ± 0.07

	Normal RV function (*n* = 6)	RV dysfunction (*n* = 7)
CEUS		
Cortex RT (s)	5.2 ± 1.0	5.0 ± 0.7
Medulla RT (s)	8.0 ± 1.3	6.2 ± 1.0^d^
IRD^c^		
RI	0.69 ± 0.09	0.73 ± 0.06
VII	0.22 ± 0.06	0.27 ± 0.06

*Note*: Data are expressed as the mean ± SD. CEUS, contrast‐enhanced ultrasonography; RT, rise time; IRD, Abbreviations: IRD, intrarenal Doppler ultrasonography; RI, resistance index; RV, right ventricular; VII, venous impedance index.

^a^ Differs significantly from the control (*P *< .05).

^b^ IRD was measured in twenty‐six dogs.

^c^ IRD was measured in twelve dogs.

^d^ Differs significantly from the normal RV function (*P *< .05).

Intrarenal Doppler ultrasonography was performed on eight dogs in the control group (66.7%), six in the stage B1 group (60.0%), and twelve in the stage B2 group (92.3%). Intrarenal Doppler ultrasonography was not performed in nine dogs due to their uncooperative behavior or tachypnea. No significant differences were observed in RI or VII among the groups (Table 2).

**FIGURE 3:**
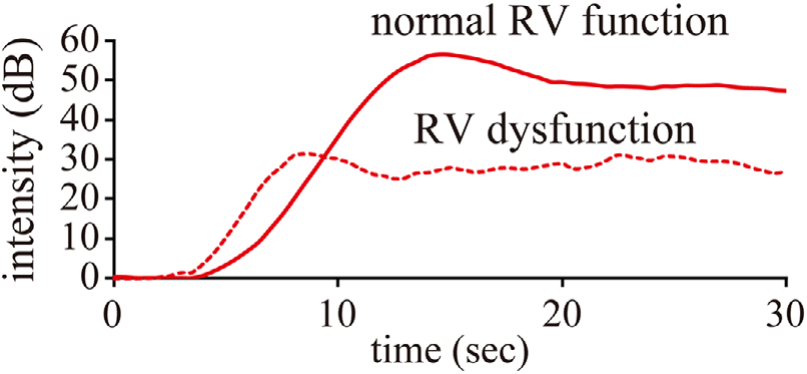
Time–intensity curve of the renal medulla obtained from representative dogs with normal right ventricular (RV) function (12‐year‐old male chihuahua) (red solid line) and RV dysfunction of stage B2 (16‐year‐old male toy poodle; red dash line). The rise time was shorter in dogs with RV dysfunction than in those with normal RV function. The X‐axis represents the time in seconds (sec), while the Y‐axis shows intensity (dB). RV, right ventricular.

The rise time of the medulla was shorter in the ACVIM stage B2 group with RV dysfunction than in dogs with normal RV function. In contrast, RT of the cortex did not significantly differ between dogs with RV dysfunction and normal RV function (Figure 3, Table 2). Furthermore, no significant differences were observed in RI or VII between dogs with RV dysfunction and normal RV function (Table 2).

### 3.4 Correlation analysis

The glomerular filtration rate did not correlate with RT of the cortex or medulla. Additionally, no correlations were observed between RT of the cortex and medulla and the left atrium to aorta root diameter ratio, normalized left ventricular end‐diastolic diameter, transmitral early diastolic flow velocity, tricuspid regurgitation velocity, RV Tei index, or stroke volume index.

## 4 DISCUSSION

To the best of our knowledge, this is the first study to examine renal perfusion by CEUS in dogs with MMVD. The results of CEUS indicated that cortical renal perfusion velocity was slower in the stage B2 group than in the control group, while medullary renal perfusion velocity was faster in the RV dysfunction in stage B2 group. On the other hand, no significant differences were observed in IRD indices between these groups. Contrast‐enhanced ultrasonography may detect changes in renal perfusion earlier than IRD, even in preclinical MMVD, as hypothesized.

In the present study, the repeatability and reproducibility of PI, an intensity‐related parameter, and WoR, a slope‐related parameter, were low. Previous reports have also shown that the repeatability and reproducibility of time‐related parameters were good, whereas those of intensity‐ and slope‐related parameters were low due to several factors, such as differences in kidney depth between individuals.^27^, ^28^ The present result agrees with previous studies. In the present study, only RT was used because it showed good reproducibility and reproducibility.

The rise time of the cortex was slower in dogs with ACVIM stage B2 than in control dogs. This result corresponds to heart failure in human patients.^14^ In heart failure patients, right heart congestion decreased after chronic heart failure treatment, and renal perfusion impairment in the cortex improved, suggesting that this improvement was due to the attenuation of renal congestion. In heart failure, decreased renal perfusion due to a reduced cardiac output and renal congestion due to RV dysfunction activate the renin‐angiotensin‐aldosterone system (RAAS) and sympathetic nervous system.^29^ Choi et al.^30^ reported a delay in renal cortical perfusion with the administration of medetomidine due to reduced cardiac output. In the present study, while reduced cardiac output was suspected as the cause of delayed cortical perfusion, stroke volume did not correlate with RT of the cortex. The activation of RAAS and the sympathetic nervous system increases angiotensin II and noradrenaline levels, resulting in vasoconstriction and reduced blood flow, particularly in the cortex.^31^, ^32^ An increase in noradrenaline due to activation of the sympathetic nervous system has been reported in dogs with preclinical MMVD.^33^ A possible loss of tissue angiotensin‐converting enzyme 2, which degrades angiotensin II, has been suggested to activate RAAS.^34^ These factors may be related to the delay observed in renal cortical perfusion in the present study. Furthermore, renal interstitial pressure may be elevated in preclinical MMVD dogs. However, hormone factors that affect RAAS, the sympathetic nervous system, and renal interstitial pressure were not examined in the present study; therefore, further research is required to elucidate the relationships between these factors and changes in renal cortical perfusion in dogs with MMVD.

In the present study, renal medullary perfusion did not significantly differ among the stages examined. Since renal medullary circulation is poorly autoregulated and is sensitive to small increases in renal interstitial pressure, the perfusion delay was more pronounced in a rat model of acute renal congestion.^35^ Furthermore, the medullary perfusion velocity was delayed in heart‐failure human patients.^14^ The present results differed from these findings. In heart failure human patients, PH, which causes RV dysfunction, and an increased right atrial pressure were observed, suggesting a perfusion impairment due to renal congestion.^14^ In dogs with MMVD, as the disease progresses, RV dysfunction develops due to the complications of PH.^36^ Since the present study targeted preclinical MMVD dogs, the severity of heart disease may be related to this difference, and medullary perfusion may be delayed in more severe cases. However, to clarify the time course of changes in renal perfusion, case‐by‐case follow‐up studies are required.

The rise time of the medulla was shorter in the RV dysfunction in the ACVIM stage B2 group than in the normal RV function group. In a dog model of increased right atrial pressure due to tricuspid regurgitation, the blood flow rate was decreased in the cortex and increased in the medulla.^37^ Although it currently remains unclear whether right atrial pressure was increased in this study, it is possible that similar changes in the distribution of renal blood flow occurred. An increased renal medullary perfusion velocity assessed by CEUS has been observed in dogs with acute kidney injury, and shunting from the renal cortex to the renal medullary area has been suggested as a cause.^17^ In human medicine, the frequency of shunting of afferent and efferent arterioles in the juxtamedullary glomerulus, which is important for maintaining medullary blood flow, increases with the severity of kidney damage.^38^ The medullary perfusion velocity may be increased due to intrarenal shunting in preclinical MMVD with RV dysfunction. The glomerular filtration rate decreased in several dogs and a correlation was not observed between the glomerular filtration rate, which reflects global renal function, and renal perfusion velocity. The fact that only the left kidney was evaluated by CEUS may be the reason why renal perfusion velocity was not associated with glomerular filtration rate. While renal perfusion velocity assessed by CEUS may reflect different changes from the glomerular filtration rate, additional studies, such as a histological investigation, are needed to confirm these points.

Significant changes were noted in the cortical perfusion index in MMVD stage B2 and the medullary perfusion index in the RV dysfunction in the MMVD stage B2 group by CEUS, but not in IRD indices among groups. This result suggests that CEUS has the capacity to detect changes in intrarenal hemodynamics earlier than IRD. In a dog model of chronic ischemic renal disease, significant changes were identified earlier in the renal perfusion index assessed by CEUS than in RI assessed by IRD.^39^ Since CEUS may quantify the perfusion of the microcirculation, it may have been possible to identify earlier changes in renal perfusion. The early detection of intrarenal hemodynamic abnormalities may be useful for the management of dogs with MMVD. However, studies involving a large number of dogs are required because true differences may not have been detected in the present study due to the small sample size.

There were some limitations that need to be addressed. Since the sample size was small, there may have been type 2 errors. While some indices in the present study showed no significant differences, the small sample size may have made the study underpowered to detect true differences. Furthermore, dogs were undergoing routine clinical treatment; therefore, the medications administered, such as pimobendan and furosemide, were not uniform. Pimobendan increases cardiac output and renal blood flow,^39^, ^40^ while furosemide decreases the circulating blood volume and renal blood flow and constricts afferent arterioles.^41^, ^42^ These effects may have an impact on renal perfusion. Medical treatment was always performed following the ACVIM consensus statement. However, it was difficult to standardize the treatment because some dogs were treated at the referring veterinary hospital. Lastly, the present study focused on RT to assess renal perfusion based on good repeatability and previous reports in heart failure human patients. The other indices, such as PI and WoR, were not evaluated due to low repeatability. If these indices could be evaluated, further findings might be obtained.

In the present study, CEUS identified decreases in cortical perfusion velocity in ACVIM stage B2 and an increase in medullary perfusion velocity in the RV dysfunction with ACVIM stage B2 group, and these changes were not detected by IRD. Collectively, the present results suggest the ability of CEUS to identify changes in renal perfusion in preclinical MMVD earlier than IRD.

## LIST OF AUTHOR CONTRIBUTIONS

### Category 1

(a) Conception and design: Kamata, Morita, Yamasaki
(b) Acquisition of data: Kamata, Morita
(c) Analysis and interpretation of data: Kamata, Morita

### Category 2

(a) Drafting the article: Kamata, Morita, Yamasaki
(b) Revising article for intellectual content: Kamata, Morita

### Category 3

(a) Final approval of the completed article: Kamata, Morita, Yamasaki

### Category 4

(a) Agreement to be accountable for all aspects of the work ensuring that questions related to the accuracy or integrity of any part of the work are appropriately investigated and resolved: Kamata, Morita, Yamasaki

## CONFLICT OF INTEREST STATEMENT

The authors declare no conflict of interest.

## PREVIOUS PRESENTATION OR PUBLICATION DISCLOSURE

This manuscript has not been published elsewhere and is not under consideration by any other journal or conference.

## REPORTING CHECKLIST DISCLOSURE

No reporting guideline checklist was used.

## References

[1] Borgarelli M , Crosara S , Lamb K , et al. Survival characteristics and prognostic variables of dogs with preclinical chronic degenerative mitral valve disease attributable to myxomatous degeneration. J Vet Intern Med. 2012;26:69‐75. 22211523 10.1111/j.1939-1676.2011.00860.x

[2] Larouche‐Lebel É , Loughran KA , Oyama MA . Echocardiographic indices and severity of mitral regurgitation in dogs with preclinical degenerative mitral valve disease. J Vet Intern Med. 2019;33:489‐498. 30793808 10.1111/jvim.15461 PMC6430891

[3] Martinelli E , Locatelli C , Bassis S , et al. Preliminary investigation of cardiovascular–renal disorders in dogs with chronic mitral valve disease. J Vet Intern Med. 2016;30:1612‐1618. 27717188 10.1111/jvim.14524 PMC5032878

[4] Damman K , van Deursen VM , Navis G , et al. Increased central venous pressure is associated with impaired renal function and mortality in a broad spectrum of patients with cardiovascular disease. J Am Coll Cardiol. 2009;53:582‐588. 19215832 10.1016/j.jacc.2008.08.080

[5] Damman K , Navis G , Smilde TDJ , et al. Decreased cardiac output, venous congestion and the association with renal impairment in patients with cardiac dysfunction. Eur J Heart Fail. 2007;9:872‐878. 17586090 10.1016/j.ejheart.2007.05.010

[6] Boorsma EM , ter Maaten JM , Voors AA , et al. Renal compression in heart failure: the renal tamponade hypothesis. JACC Hear Fail. 2022;10:175‐183. 10.1016/j.jchf.2021.12.005 35241245

[7] Mullens W , Abrahams Z , Francis GS , et al. Importance of venous congestion for worsening of renal function in advanced decompensated heart failure. J Am Coll Cardiol. 2009;53:589‐596. 19215833 10.1016/j.jacc.2008.05.068 PMC2856960

[8] Iida N , Seo Y , Sai S , et al. Clinical implications of intrarenal hemodynamic evaluation by Doppler ultrasonography in heart failure. JACC Hear Fail. 2016;4:674‐682. 10.1016/j.jchf.2016.03.016 27179835

[9] Seo Y , Iida N , Yamamoto M , et al. Doppler‐derived intrarenal venous flow mirrors right‐sided heart hemodynamics in patients with cardiovascular disease. Circ J. 2020;84:1552‐1559. 32669529 10.1253/circj.CJ-20-0332

[10] Seo Y , Nakatsukasa T , Sai S , et al. Clinical implications of organ congestion in heart failure patients as assessed by ultrasonography. Cardiovasc Diagn Ther. 2018;8:57‐69. 29541611 10.21037/cdt.2017.07.05 PMC5835652

[11] Chiba H , Seo Y , Sai S , et al. Renoprotective effects of tolvaptan in hypertensive heart failure rats depend on renal decongestion. Hypertens Res. 2019;42:319‐328. 30559403 10.1038/s41440-018-0169-3

[12] Morita T , Terukina H , Yamasaki M . Evaluation of intrarenal blood flow using pulsed‐wave Doppler ultrasonography in dogs with myxomatous mitral valve disease. J Vet Intern Med. 2024; Under review.

[13] Morita T , Terukina H , Kobayashi S , et al. Intrarenal venous flow analysis allows for more detailed stratification of the prognosis of dogs with myxomatous mitral valve disease. In: 119th Conference of Japanese Society of Veterinary Cardiology. 2023.

[14] Komuro K , Shimazu K , Koizumi T , et al. Demonstration of improved renal congestion after heart failure treatment on renal perfusion imaging with contrast‐enhanced ultrasonography. Circ Reports. 2019;1:593‐600. 10.1253/circrep.CR-19-0024 PMC7897700 33693105

[15] Dong Y , Wang W , Cao J , et al. Quantitative evaluation of contrast‐enhanced ultrasonography in the diagnosis of chronic ischemic renal disease in a dog model. PLoS One. 2013;8:e70337. 23936410 10.1371/journal.pone.0070337 PMC3731349

[16] Lee G , Jeon S , Lee SK , et al. Quantitative evaluation of renal parenchymal perfusion using contrast‐enhanced ultrasonography in renal ischemia‐reperfusion injury in dogs. J Vet Sci. 2017;18:507‐514. 28385013 10.4142/jvs.2017.18.4.507 PMC5746444

[17] Mannucci T , Lippi I , Rota A , et al. Contrast enhancement ultrasound of renal perfusion in dogs with acute kidney injury. J Small Anim Pract. 2019;60:471‐476. 31012121 10.1111/jsap.13001

[18] Keene BW , Atkins CE , Bonagura JD , et al. ACVIM consensus guidelines for the diagnosis and treatment of myxomatous mitral valve disease in dogs. J Vet Intern Med. 2019;33:1127‐1140. 30974015 10.1111/jvim.15488 PMC6524084

[19] International Renal Interest Society . IRIS staging of CKD (modified 2023). 2023; Available at: http://www.iris‐kidney.com/guidelines/staging.html

[20] Reinero C , Visser LC , Kellihan HB , et al. ACVIM consensus statement guidelines for the diagnosis, classification, treatment, and monitoring of pulmonary hypertension in dogs. J Vet Intern Med. 2020;34:549‐573. 32065428 10.1111/jvim.15725 PMC7097566

[21] Cornell CC , Kittleson MD , Torre PD , et al. Allometric scaling of M‐mode cardiac measurements in normal adult dogs. J Vet Intern Med. 2004;18:311‐321. 15188817 10.1892/0891-6640(2004)18<311:asomcm>2.0.co;2

[22] Morita T , Nakamura K , Osuga T , et al. The repeatability and characteristics of right ventricular longitudinal strain imaging by speckle tracking echocardiography in healthy dogs. J Vet Cardiol. 2017;19:351‐362. 28739084 10.1016/j.jvc.2017.05.001

[23] Boon JA . Veterinary echocardiography. 2nd ed. Wiley‐Blackwell; 2011.

[24] Peng S , Ding H , Fu T , et al. Savitzky‐Golay filter based contrast‐enhanced ultrasound quantification in hepatic tumors: methodology and its correlation with tumor angiogenesis. Clin Hemorheol Microcirc. 2016;73:271‐282. 10.3233/CH-180432 30103307

[25] Terukina H , Morita T , Yamasaki M . The repeatability, reproducibility, and influencing factors of intrarenal Doppler ultrasonography in dogs without heart disease. Vet Radiol Ultrasound. 2023;64:337‐344. 36447301 10.1111/vru.13194

[26] Nisa K , Lim SY , Shinohara M , et al. Repeatability and reproducibility of quantitative contrast‐enhanced ultrasonography for assessing duodenal perfusion in healthy dogs. J Vet Med Sci. 2017;79. 10.1292/jvms.17-0174 PMC5627333 28781327

[27] Stock E , Duchateau L , Saunders JH , et al. Repeatability of contrast‐enhanced ultrasonography of the kidneys in healthy cats. Ultrasound Med Biol. 2018;44:426‐433. 29174044 10.1016/j.ultrasmedbio.2017.09.019

[28] Liu DJX , Hesta M , Stock E , et al. Renal perfusion parameters measured by contrast‐enhanced ultrasound in healthy dogs demonstrate a wide range of variability in the long‐term. Vet Radiol Ultrasound. 2019;60:201‐209. 30276919 10.1111/vru.12690

[29] Pouchelon JL , Atkins CE , Bussadori C , et al. Cardiovascular‐renal axis disorders in the domestic dog and cat: a veterinary consensus statement. J Small Anim Pract. 2015;56:537‐552. 26331869 10.1111/jsap.12387 PMC4584495

[30] Choi SY , Jeong WC , Lee YW , et al. Contrast enhanced ultrasonography of kidney in conscious and anesthetized beagle dogs. J Vet Med Sci. 2016;78:239‐244. 26412201 10.1292/jvms.15-0199 PMC4785112

[31] Steinhausen M , Ballantyne D , Fretschner M , et al. Different responses of cortical and juxtamedullary arterioles to norepinephrine and angiotensin II. Kidney Int Suppl. 1990;30:S55‐59. 2259077

[32] Yamamoto T , Hayashi K , Matsuda H , et al. In vivo visualization of angiotensin II‐ and tubuloglomerular feedback‐mediated renal vasoconstriction. Kidney Int. 2001;60:364‐369. 11422773 10.1046/j.1523-1755.2001.00808.x

[33] Uechi M , Shimizu A , Mizuno M . Heart rate modulation by sympathetic nerves in dogs with heart failure. J Vet Med Sci. 2002;64:1023‐1029. 12499688 10.1292/jvms.64.1023

[34] Hammond HH , Ames MK , Domenig O , et al. The classical and alternative circulating renin‐angiotensin system in normal dogs and dogs with stage B1 and B2 myxomatous mitral valve disease. J Vet Intern Med. 2023;37:875‐886. 36951394 10.1111/jvim.16687 PMC10229370

[35] Komuro K , Seo Y , Yamamoto M , et al. Assessment of renal perfusion impairment in a rat model of acute renal congestion using contrast‐enhanced ultrasonography. Heart Vessels. 2018;33:434‐440. 29027577 10.1007/s00380-017-1063-7

[36] Nakamura K , Morita T , Osuga T , et al. Prognostic value of right ventricular Tei index in dogs with myxomatous mitral valvular heart disease. J Vet Intern Med. 2016;30:69‐75. 26789419 10.1111/jvim.13820 PMC4913668

[37] Barger AC . Renal hemodynamic factors in congestive heart failure. Ann N Y Acad Sci. 1966;139:276‐284. 5230273 10.1111/j.1749-6632.1966.tb41202.x

[38] Takazakura E , Sawabu N , Handa A , et al. Intrarenal vascular changes with age and disease. Kidney Int. 1972;2:224‐230. 4657923 10.1038/ki.1972.98

[39] Pagel PS , Hettrick DA , Warltier DC . Influence of levosimendan, pimobendan, and milrinone on the regional distribution of cardiac output in anaesthetized dogs. Br J Pharmacol. 1996;119:609‐615. 8894186 10.1111/j.1476-5381.1996.tb15716.x PMC1915696

[40] Kanno N , Kuse H , Kawasaki M , et al. Effects of pimobendan for mitral valve regurgitation in dogs. J Vet Med Sci. 2007;69:373‐377. 17485924 10.1292/jvms.69.373

[41] Sarraf M , Masoumi A , Schrier RW . Cardiorenal syndrome in acute decompensated heart failure. Clin J Am Soc Nephrol. 2009;4:2013‐2026. 19965544 10.2215/CJN.03150509

[42] Francis GS , Siege RM , Goldsmith SR , et al. Acute vasoconstrictor response to intravenous furosemide in patients with chronic congestive heart failure. Ann Intern Med. 1985;103:1‐6. 2860833 10.7326/0003-4819-103-1-1

